# The role of take-over cue informativity in interrupted take-over requests in a semi-automated driving scenario

**DOI:** 10.1038/s41598-026-36614-y

**Published:** 2026-01-20

**Authors:** Alexander Berger, Nicole Damm, Martin Baumann, Markus Kiefer

**Affiliations:** 1 Department of Psychiatry, Section for Cognitive Electrophysiology, Ulm University, Ulm, Germany; 2 Department of Psychology, Ulm University, Ulm, Germany

**Keywords:** Semi-automated driving, Take-over request, Task cuing, Practice effects, Mathematics and computing, Neuroscience, Psychology, Psychology

## Abstract

In semi-automated driving, take-over requests (TOR) signal the driver to take over control, if the car’s driving system is not able to handle a particular traffic situation autonomously. While previous research indicated that providing informative TORs aids take-over behavior, related experimental research on task cuing suggested that informative cues might also be harmful, if interrupted by another task. Hence, the present study tested whether the advantage of informative TORs diminishes if after a TOR cue an interrupting task is presented. This should resemble a scenario, where drivers are about to take over control in response to a TOR, but then are distracted, for example by an incoming phone call. To this end, we presented participants with videos of a semi-automated driving situation, where task-specific informative or generic, non-informative TOR cues indicated an upcoming take-over action. Crucially, in half of the trials, this TOR cue was interrupted. Results showed informative TOR cues to generally improve take-over performance, but the interrupting task to reduce the advantage of providing informative TORs. However, this effect took some time until it reached its maximum and vanished with further practice. In sum, the present study identified interrupting, distracting information and practice as factors moderating the effectiveness of TOR cues, which demand further consideration in the research of semi-automated driving.

**Supplementary Information:**

The online version contains supplementary material available at 10.1038/s41598-026-36614-y.

## Introduction

The emergence of new automation technologies creates new demands for human operators while interacting with them. As a consequence, a multitude of yet-to-be-explored psychological effects arise in men-machine interaction. Take for example the field of semi-automated driving, where the driving system operates the car autonomously most of the time but requires the human driver to take over control, if it fails to handle a particular situation^1^. According to the taxonomy of the Society of Automotive Engineers (SAE), this refers to a level 3 automation (hereafter referred to as semi-automated driving)^1^. As the driving system performs the driving task mostly autonomously, it allows the human driver to engage in other, so-called non-driving related tasks (NDRT)^2^ like reading a newspaper, doing an office task or watching a video^3–6^. However, if the semi-automated driving system fails to handle a particular driving situation, the human driver must take over control of the driving task within a given time interval. This need to take over control (*take-over request*; TOR) is usually signaled by a cue^7,8^. As failing to appropriately react to such TORs can clearly be dangerous, the factors which favor or disfavor such take-over behavior were previously extensively studied, for reviews see^8,9^.

### Take-over requests in semi-automated driving

To this end, the typical design of investigating TOR effectiveness involves a setting in which the semi-automated driving system operates the car autonomously, and the human driver then receives a TOR, measuring subsequent take-over performance (often in terms of take-over latency)^9^. Before TOR onset (i.e., during automated driving), the human operator likely loses situation awareness regarding the driving situation^10–12^, with high situation awareness being required to be able to quickly and accurately react in men-machine interaction^13–15^. This is especially the case, when the human driver engages in NDRTs, as was for example demonstrated for conversations with a passenger, phone calls and smartphone interactions^6,16,17^. This further underscores the importance of designing TORs which efficiently get the human operator back in control, i.e., re-gain high situation awareness.

With that regard, studies observed that multimodal TOR cues, e.g., combining vision and audition, are beneficial compared to unimodal ones^7,18,19^ and that more time available to react to the TOR results in more accurate take-over performance^7,9^. Furthermore, cuing the required action^7^ as well as providing more information regarding the hazard demanding the take-over action^20^ accelerated take-over time and improved situation awareness indexed by eye movements, respectively. Considering the role of distraction due to NDRTs, NDRTs were shown to impair take-over performance, in terms of longer take-over latencies^21^ and delayed behavior related to situation awareness, e.g., delayed mirror checking^3–5^.

Thus, distraction *before* TOR onset, for example due to NDRTs, deteriorated take-over behavior, with a possible approach to reduce such detrimental effects being to provide more informative TOR cues. However, here we wanted to investigate a scenario being unexplored by these previous studies: a driver receives a TOR cue but then is distracted in the moment of initiating some take-over action. For example, imagine a semi-automated driving system operating in autonomous mode. After some time, it encounters a traffic situation exceeding its system capacities and initiates a TOR, asking the human driver to take over control manually. During the process when the driver re-orientates to the driving situation, however his/her phone is ringing, causing distraction. As the phone call likely captures attentional resources and could initiate actions related to the phone (muting the phone, accepting or declining the phone call), the take-over behavior might consequently suffer. Accordingly, in the present work, we specifically investigated the impact of distraction *after* TOR onset on take-over performance.

### Task cuing effects related to TORs

The relevance of this specific research question can be inferred from cognitive-psychological experiments on task cuing. In these experiments, task cues providing more specific and straightforward information regarding the upcoming task (thus being similar to informative TORs) were subject to inhibitory processes, if interrupted by an interfering task^22,23^, for a general overview of task interruptions see^24^. These inhibitory mechanisms were investigated by means of so-called *cue-only trials*^25–28^. While in the classical task cuing paradigm, the task cue indicating the to-be-performed task is always followed by that respective task^29,30^, in cue-only trials only the task cue is presented, omitting the subsequent task. This allows investigating the effect of mere task preparation on subsequent processing.

Task switching research demonstrated that processing a task cue activates a corresponding cognitive task representation – called *task set*^30^. This prepared task set facilitates later task performance, and more so when the link between cue and task is more transparent (for example, by providing the first letter of the task as cue)^31–34^. However, when the prepared task set must not be executed – i.e., a cue-only trial is presented, and participants must switch after cue presentation to another task – the cued task set is inhibited, impeding a later return to this task^22,28,35^. Interestingly, task cue transparency resulted in stronger inhibitory action, hence showing detrimental effects on the return to the cued task after interruption. For instance, the task was either cued by the first letter of the associated decision category (e.g., the letter “R” for a round, german “Rund” decision, or the letter “B” for a living, german “Belebt” decision), forming a transparent link between cue and task, or by the first letter of the alternative decision category (“R” → “Belebt”; “B” → “Rund”), forming cues lacking any direct link with the associated task. For the former, transparent cue-task association, larger inhibitory effects were observed following cue-only trials^22,23,28^, but also see^35^, for similar inhibitory effects following different cue types.

Thus, translating these findings into the field of semi-automated driving, an informative TOR cue providing more details about the required take-over action might be less effective, if during the process of taking over interrupting, distracting information is presented. Note that this poses a different question compared to previous research investigating the influence of distracting NDRTs in the context of TORs. There, the driver disengages from the driving task to perform some NDRT and is later forced to return to the driving task when a TOR occurs. In contrast, in the scenario investigated in the present study the driver is already in the process of returning to the driving task as reaction to a TOR but then becomes interrupted by a distracting task.

### Relevance of practice for task cuing effects and TOR effectiveness

Here, we aimed to investigate the cognitive-psychological mechanisms at play during such a semi-automated driving scenario and therefore conducted an in-lab experiment inspired by previous task cuing research. This allows decent experimental control for testing whether interrupting a take-over action in response to an informative TOR cue is especially harmful. This prediction is suggested by larger inhibitory effects following transparent cue-only trials in task cuing research^22,23,28^. Though, the ecological validity of such an experimental approach is clearly limited: While providing many trials in which a TOR cue is either interrupted by a distracting task or remains uninterrupted allows precisely measuring the impact of this interruption, it does not capture the actual driving situation in all aspects. There, the human operator likely has no prolonged experience with the interruption of TOR cues, and the first experience of a TOR situation might already be a safety-critical one (for further limitations in ecological validity of the present design, see the *Discussion*). However – when practicing TORs was possible – take-over actions involved less breaking behavior in a driving simulator^19^ and were performed more quickly in actual on-the-road driving^36^ with repeated exposure to such events, suggesting that take-over behavior becomes more efficient with practice. To specifically look at the impact of interrupting TORs at little practice in the present experimental approach based on many trials, we adopted *effect course analysis*^37^, which allows inspecting the course of experimental effects on a continuous time scale within a session and thus enables investigating how practice changes the effect of interruption on TORs. This type of analysis already revealed the effects of cue-only trials to be reduced after extended practice in a task cuing context^22,23,37^, thus emphasizing the relevance for such an analysis also from a cognitive-psychological perspective.

### The present study

**Fig. 1:**
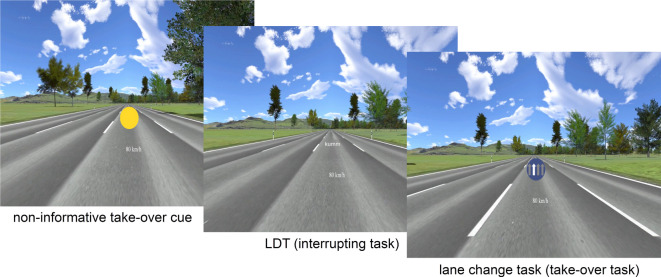
Example trial in this experiment. Participants watch a video of a car driving on a road from an ego perspective. The car can drive at four given speeds (here: 80 km/h) and on one of four lanes (here: middle-right lane). After a jittered duration, a cue is presented, reflecting a take-over request (first picture). In this case, the cue is non-informative, indicating both possible take-over tasks with the same probability. Afterwards, either the take-over task directly follows the cue, or an interrupting lexical decision task (LDT, second picture) follows. After having performed the interrupting task (if presented), the take-over task is shown, indicated by a stimulus signaling the to-be-performed operation. In the present example (third picture), a stimulus for the lane change task is shown, signaling the participant that she/he has to change to the middle-left lane. From the current perspective of that participant, this corresponds to a change of one lane to the left, and he/she has to press the left button.

Taken together, the present study was designed to investigate the influence of task cues representing a TOR on the performance of this cued take-over behavior, when the process of reacting to the TOR is interrupted by a distracting task. With this regard, it combined cognitive-psychological with applied research methods and explored the driving behavior in a controlled, experimental environment – i.e., a model scenario for semi-automated driving. Thus, the goal of the present study is to identify a potential safety-critical mechanism using cognitive-psychological research methods, which in the end may be transferred into a more applied research context. Participants watched videos of a car driving on a road as a proxy for a semi-automated driving situation. During this situation, they were cued to take over-control of the semi-automated car, with this cue either indicating the to-be-performed lane or speed change task or being uninformative regarding which of these two tasks will be presented. Accordingly, non-informative cues signaled the occurrence of a task, but not its identity – being comparable to a generic warning signal. Effectiveness of TORs was measured by response time (RT) and error rate (ER) differences between informative and non-informative cues. Crucially, in half of the trials, the cue was followed by a lexical decision task (LDT; decision whether a letter string is a meaningful word, or a pronounceable, but meaningless pseudoword), which served as proxy for various distracting, interrupting information, like for example an incoming phone call. Participants had then to switch to the LDT and perform this task before returning to the speed/lane change task (the LDT is therefore also referred to as *interrupting* task, the driving-related speed/lane change tasks are conjointly referred to as *take-over* task). Note that in the present experiment, participants needed to always respond to the interrupting task before returning to the take-over task. In real driving, the take-over action likely has highest priority and probably will be executed prior to responding to any interrupting tasks. Nevertheless, it cannot be entirely ruled out that a driver responds to a phone call or a message on the smartphone in an impulsive fashion after having received a TOR. Thus, here the distraction might have a more severe impact compared to an actual driving scenario, where there is no need to react to the distraction. However, this reaction to the interrupting LDT was necessary in our experimental scenario, as participants could have otherwise deliberately ignored the LDT. Even when a distraction does not trigger actions towards it, it likely captures attentional processes^38–40^ and/or can result in cognitive effort due to inhibiting any reflex-like reactions^41^. Thus, we think that the LDT may serve as a valid proxy for the possible harmful effects of distracting information in a driving context, and more specifically for the case when drivers react to a distraction occurring after a TOR. An example trial is shown in Fig. 1.

In general, we expected better performance in the take-over task if it was preceded by an informative compared to a non-informative TOR cue. However, we hypothesized this effect to be moderated by the presence of the interrupting task, here LDT. If an interrupting task was presented, we expected the informativity effect to be reduced or even reversed reflecting an inhibition of the cued task set when switching to the interrupting task^22,28,35^. We will focus on the modulation of the informativity effect, i.e., the difference between informative and non-informative cues, and not the specific RT level following these two cue types, as the general influence of the interrupting task on take-over task performance is difficult to predict. The LDT may be generally experienced distracting, if participants consistently only prepare for the cued task, thus hampering performance following such an interruption. Alternatively, the introduction of a frequent interrupting task may encourage participants to prepare for both tasks, resembling a dual-task situation^42,43^, which might attenuate performance if the first task is lacking (no interrupting LDT presented).

This experimental design is expected to give insights into the cognitive processes which are involved when a distraction occurs after a TOR. As in real driving the driver is likely not exposed to as many TORs as in the present experiment, and the first TOR might already be a safety-critical one, here we specifically also looked at effects after little practice. To this end, we applied effect course analysis, and hypothesized the reduction/reversal of the cue-informativity effect by the interrupting LDT to be most pronounced at the beginning of the experiment, i.e., to decrease with practice.

## Results

### Performance in the interrupting task

A paired t-test on mean RTs in the interrupting LDT showed a significant RT difference, if it was preceded by an informative (abbreviated as “info”) compared to a non-informative cue (“non-info”), *t*(44) = 6.48, *p* < .001. Mean RTs in the interrupting task were 787 ms if preceded by an informative cue, and 756 ms if preceded by a non-informative cue (Cohen’s *d* = 0.97). Thus, LDT performance was slowed, if the preceding cue indicated the identity of the upcoming take-over task. A corresponding analysis on mean ERs did not reveal a significant difference, *t*(44) = 0.81, *p* = .425 (mean ER[info] = 0.039, mean ER[non-info] = 0.034, Cohen’s *d* = 0.12).

### Performance in the take-over task

**Fig. 2:**
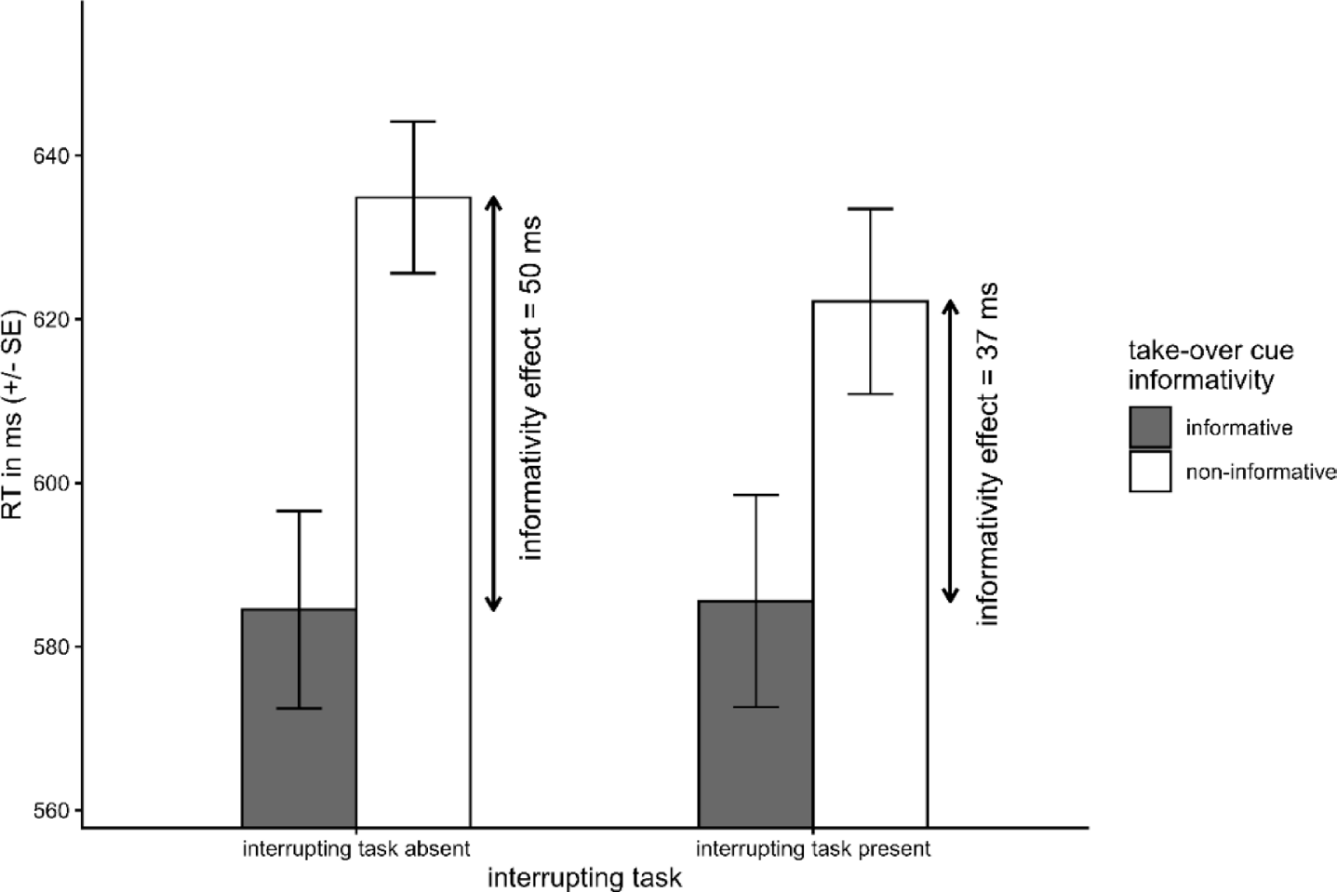
Mean RTs (plus associated standard error) in the take-over task. Shown are mean RTs separately for each cue-informativity condition and as a function of the absence or presence of the interrupting task (interrupting task absent vs. interrupting task present). The cue-informativity effect (“informativity effect”; shown alongside the arrows) was reduced following the interrupting task.

A repeated-measures ANOVA on mean RTs in the take-over task revealed a significant main effect of cue-informativity, *F*(1,44) = 64.24, *p* < .001. On average, RTs following informative cues were around 40 ms faster (mean RT[info] = 585 ms, mean RT[non-info] = 628 ms, Cohen’s *d* = − 0.57). Hence, providing an informative cue accelerated responding. The main effect of interrupting task did not reach significance, *F*(1,44) = 1.17, *p* = .285; there was no significant RT difference regarding whether the take-over task was preceded by an interrupting LDT (mean RT = 604 ms) or not (mean RT = 610 ms, Cohen’s *d* = − 0.08). However, the interaction of both factors reached significance, *F*(1,44) = 5.33, *p* = .026. The cue-informativity effect was reduced following an interrupting task (mean informativity effect = 37 ms, Cohen’s *d* = − 0.48) compared to when the take-over task directly followed the take-over cue (50 ms, Cohen’s *d* = -0.65), see Fig. 2. Note that the cue-informativity effect was significant in both conditions (both *p*s < 0.001, for corresponding post hoc tests see *Supplementary Material G*).

A corresponding ANOVA on mean ERs did not show any significant main effect, both *F*s < 0.82, both *p*s > 0.370. Neither did informative cues significantly reduce errors (mean ER[info] = 0.008, mean ER[non-info] = 0.009, Cohen’s *d* = − 0.08), nor did the presence of the interrupting task significantly increase errors (mean ER[interrupting task present] = 0.010, mean ER[interrupting task absent] = 0.007, Cohen’s *d* = 0.16). Likewise, while the cue-informativity effect was descriptively larger when the interrupting task was absent (Cohen’s *d* = − 0.25), compared to its presence, where it was descriptively reversed (Cohen’s *d* = 0.08), the corresponding interaction did not reach significance, *F*(1,44) = 3.08, *p* = .086.

### Effect course analysis of response times in the take-over task

**Fig. 3:**
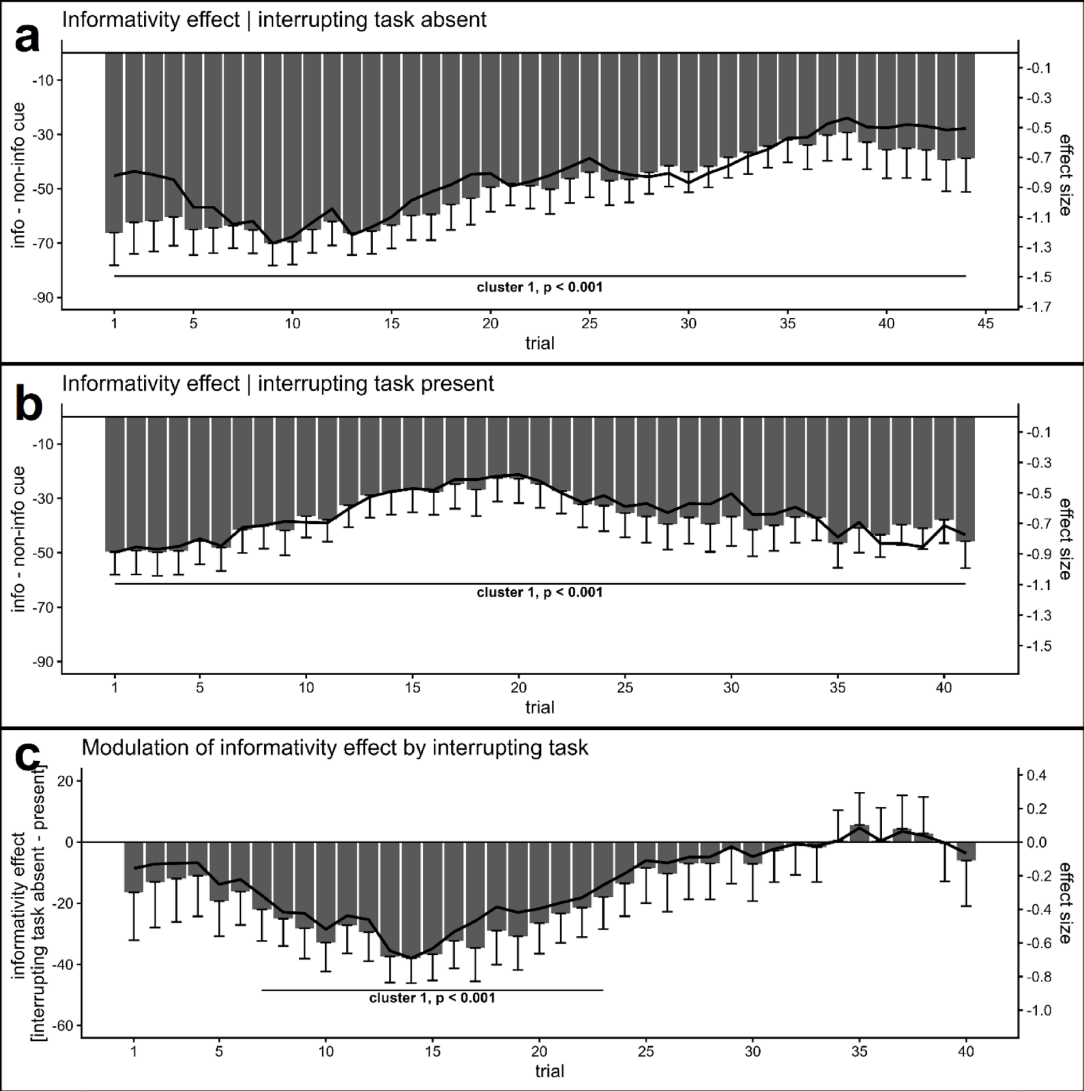
Effect course analyses on take-over task RTs. The bars show the RT difference (plus associated SEs as whiskers) between informative and non-informative cues separately for trials where no interrupting LDT was presented, and which were preceded by the interrupting task (panel a and b, respectively). The black line shows the corresponding effect size of this difference (Cohen’s *d*). Panel c shows the difference between these two effect courses, i.e., how the informativity effect is modulated by the absence/presence of the interrupting task. A significant modulation was only observed in the first half of the experiment, after some initial practice.

To track whether the observed interaction of cue-informativity with the presence/absence of the interrupting task changed with practice, we performed effect course analyses on RTs in the take-over task. Effect course analysis of the cue-informativity effect, if no interrupting task was presented, showed one significant cluster, spanning across the whole analyzed time window, *T* = − 242.92, *p* < .001, trials 1–44. As can be seen in Fig. 3a, the informativity effect (faster responses to informative cues), was maximal at the beginning of the experiment, decreased with practice, but remained significant. For the corresponding analysis in trials, where the take-over task was preceded by the interrupting LDT, a significant cluster spanning across the whole analyzed time period was found as well, *T* = − 164.83, *p* < .001, trials 1–41 (see Fig. 3b). Of particular interest, regarding the modulation of the informativity effect by the absence/presence of the interrupting task, i.e., the difference of these two effect courses, one significant cluster was observed. The informativity effect was stronger, if TOR-cues were not interrupted, but only around one-fifth to one-half of the experiment, *T* = − 51.65, *p* < .001, trials 7–23. Hence, the modulation of the informativity effect by the interrupting task took some time to become significant and vanished with further practice (Fig. 3c).

For the sake of consistency, we also performed effect course analyses on take-over task ERs, although for mean ERs no significant effect was observed. These analyses are reported in *Supplementary Material A* and show a similar course of the interaction effect as the corresponding analysis on RTs.

## Discussion

The main goal of the present study was to test whether the advantage of providing informative TOR cues during semi-automated driving vanishes, if in the moment of taking over control the driver is interrupted by a distracting task or information. Such possible paradoxical effects of cuing take-over actions, i.e., a worse performance following an informative cue after an interruption, were inferred from related cognitive-psychological research. These studies indicated stronger inhibition effects following transparent in contrast to arbitrary cues, if the cue was followed by an interrupting task other than the cued one^22,23,28^. We transferred this setting into the framework of a cognitive-psychological experiment, which should resemble for example an incoming phone call in the moment the driver is about to take over control in response to a TOR. Hence, the present study was aimed to give insights into the cognitive mechanisms at play during an interruption or distraction occurring *after* TOR onset – a scenario to the best of our knowledge previously not being considered in the context of semi-automated driving.

Two main findings were obtained. First, in contrast to above-outlined previous task cuing research, in the present study informative cues consistently accelerated take-over task RTs compared to non-informative ones, irrespective of the presence/absence of an interrupting task (see Figs. 2 and 3a and *b*). Thus, in accordance to previous studies in the context of semi-automated driving^7,20^, the results of the present study demonstrate the superiority of informative cues for TORs. Second, we nonetheless observed a reduction in the cue-informativity effect if the TOR cue was interrupted by the LDT. Consequently, being interrupted during the process of taking over was harmful and should be avoided if possible.

While an interruption occurring after a take-over cue deteriorated take-over performance, the performance following informative cues did not became worse than for non-informative cues, which the assumption of a pure inhibitory mechanism would predict, cf^41^. Accordingly, in this regard, results of the present study differed from those of previous task cuing studies, which observed an inhibition of cued task sets, when after the cue a task other than the cued one followed^22,23,28^. Consequently, possible inhibitory mechanisms in the present study must have been less pronounced compared to that previous work. In these previous studies, when the cue was interrupted by a different task, it was evident to the participants that the cued task had not to be executed later after the interrupting task. In contrast, here the cued take-over task always had to be performed following the interrupting task, which appears natural given the high priority of TORs in semi-automated driving. This need to later execute the cued take-over task likely has reduced the engagement into inhibitory processes^37^. Nevertheless, presenting an interrupting task reduced the effectiveness of informative cues, and the interrupting task thus interfered with (preparation for) the take-over task. For a more detailed discussion about the cognitive mechanisms underlying the observed result pattern, and why the presence/absence of the interrupting task mainly affected the non-informative cue condition, see the *Supplementary Material.* Crucially, additional drift-diffusion model analyses^44,45^ presented in the *Supplementary Material* clearly demonstrated a detrimental effect of the interrupting task on preparation for the take-over task following informative cues. In summary, it is advisable to provide informative TOR cues. Furthermore, if one wants to take full advantage of informative TOR cues, it should be ensured that (processing of) the cued TOR is not interrupted by distracting information, e.g., by blocking incoming phone calls or muting music. This also relates to previous findings in semi-automated driving, which showed the detrimental effects of NDRTs to be reduced, if the driver is locked-out of the NDRT with TOR onset^46^.

Considering the practical relevance of the observed findings, we reasoned that effects of an interrupting task after little practice are of particular relevance, as drivers usually have no extended practice with TORs. In this regard, the significant reduction of the cue informativity effect after an interrupting task was only observed after some initial practice and not at the end of the experiment, where further practice eliminated this modulation (Fig. 3c). The absence of this modulation at the end of the experiment is not surprising, as processing likely was optimized with practice, thereby reducing interference between different tasks, e.g., task sets may have become shielded from interference^47,48^, or integrated into one overarching set^43^.

In contrast, the reason for the initial lack of this modulation is more difficult to explain. Previously, we observed hints that how participants distributed cognitive resources between two tasks changed with practice^37^. Moreover, the inhibition of task sets depends on contextual factors^22,49–51^, and might not be always engaged, but only if it aids task performance. Accordingly, inhibition of the task set of the take-over task was presumably engaged after a few trials, when interference between the take-over task and the LDT increased during the conjoint optimization of processing the sequence of two tasks. As such an optimization of task processing might be specific to the cognitive-psychological experimental nature of the current study, where the task sequence was somehow predictable, it remains open whether such practice-dependence transfers to an on-road driving setting. Nonetheless, as previous driving studies also showed take-over behavior to improve with practice in a driving simulator^19^ and in on-road driving^36^, these findings highlight the theoretical and methodological relevance of inspecting practice-related changes. As the practice state, in which human operators are, turned out to be indicative of their performance, the level of practice should not be neglected if one wants to predict how distracting information affects human behavior in men-machine interaction, moving beyond the analysis of mean behavior^37,52^. Moreover, practicing take-over actions in a controlled environment could prevent dangerous, inefficient behavior as well as misunderstandings in critical driving situations, and further research could investigate whether a practice environment for such situations is feasible.

As outlined above, the present in-lab realization of a semi-automated driving situation from a cognitive-psychological perspective cannot be directly compared to a real driving situation. However, it provided insights into the cognitive fundamentals of the process of switching to an action which was interrupted. As taking over control in semi-automated driving likely constitutes a complex, multi-faceted process^53^, in-lab investigations under controlled conditions can reveal information regarding factors which need to be considered in actual on-road behavior. In that sense, the present study can stimulate more applied upcoming research with a higher ecological validity. For example, in semi-automated driving on the road, distraction is likely less predictable compared to the present work, both temporally and regarding its content. Here, the interrupting LDT was a clearly defined task, while in actual driving distractions can take various forms (e.g., incoming phone call, passenger talking in, loud passage in a song). Thus, in an on-road setting, a possible distraction is (at least) less predictable and therefore is likely even more disruptive. A similar reasoning holds for the presentation of TORs, which occurred on each trial in the present study. In driving, such TORs are hardly to predict, especially if one engages in NDRTs which reduce situation awareness^6,16,17^. Taken together, as participants could have become familiar with the interrupting situation in the present study, they probably developed strategies to handle this distraction (compare the results of the effect course analysis). The here observed effects are therefore expected to be even more pronounced in an on-road setting, if drivers react to the distraction before returning to the TOR task. For the case when a distraction only captures attention but does not initiate any action by the driver – in contrast to the present study, where each distraction required a response – the impact of distraction might be reduced, but most likely will still be more harmful compared to the complete absence of any distraction or interruption.

A further limitation in ecological validity of the present study is the choice of purely visual TORs. Although all tasks were visual in the present study, and it was therefore unlikely that participants have missed the TOR cue, in real semi-automated driving multimodal cues are typically used so that drivers do not miss a purely visual cue if attention is directed elsewhere, e.g. when reading a newspaper, cf^7^. As only tasks with visual stimuli were used, the present study cannot provide specific information on how the overlap between TOR modality and modality of the distracting task influences TOR performance. This relationship also cannot be easily inferred from previous studies, as research on the overlap between TOR modality and modality of distracting information (e.g., NDRTs) revealed mixed findings^6,46,54^. Future studies could investigate whether and how multimodal distracting tasks affect take over behavior signaled by multimodal TOR cues.

On top, to gain further insights into how distraction or interruption influences TOR behavior, it is also advisable to include additional measures than RT (and ER) in further applied work. For example, braking behavior and mirror checking might reveal additional information about the level of situation awareness during the process of taking over beyond the total time required to take-over control^3,4^. Moreover, as suggested by the practice-dependence of the observed effects, upcoming work could also test more advanced drivers compared to the relatively young sample recruited in the present work, to test whether distraction becomes less disruptive with increased driving experience (especially in the context of semi-automated driving).

To summarize, the present work investigated the impact of interruptions occurring *after* TOR onset, to test whether the process of taking over suffers if distracting information is presented when drivers are about to initiate a take-over action. This scenario realized in a cognitive-psychological experiment should for instance resemble the situation of an incoming phone call immediately after TOR onset. Although more applied research is required to corroborate the present in-lab findings in a real semi-automated driving situation, the present study has some clear implications. Providing informative TOR cues during a semi-automated driving scenario revealed robust benefits, even in the context of an interrupting task, and thus should be preferred in semi-automated driving. However, the interrupting task reduced this cue-informativity benefit. Thus, if possible, interrupting or distracting information should be suppressed when the system provides a TOR, for example by muting incoming phone calls. Moreover, TOR effects in the context of an interrupting task became less interference-prone with practice. Accordingly, a further promising research avenue could be the design of a practice environment for take-over actions, as practice changed how efficient take-over situations were managed. In sum, the present combination of cognitive-psychological and applied research methods provided information about how TORs might be processed on a cognitive level, possibly also enriching the understanding of how human operators behave during actual semi-automated driving. Future studies could investigate the effects of interrupting tasks on TORs with different levels of informativeness in on-the road semi-automated driving.

## Method

This experiment was pre-registered (https://osf.io/eb5f4) and data, as well as analysis scripts are publicly available at: https://osf.io/hg73v/. The local ethics committee of Ulm University approved this study, and all participants gave written informed consent.

### Participants

*N* = 47 healthy subjects without any neurological or psychological disorders were recruited. This sample size was chosen to achieve a power of $\:1-\:\beta\:=0.95\:(\alpha\:=0.05)$ for detecting an effect size $\:{\eta\:}_{p}^{2}=0.074$ or larger, which was previously observed for similar effects of cue-only trials following an interfering LDT in a task switching context^22^. Participants were required to possess a driving license. *N* = 1 participant was excluded as an initial screening for color blindness (Ishihara color test; assessed from https://www.colorlitelens.com/ishihara-test.html) indicated that this person was color blind, which might affect perception of driving scenes and stimuli. Note that this exclusion criterion was not explicitly pre-registered, as it was performed independent of data analysis. Furthermore, *n* = 1 participant was excluded due to extraordinary long response times (RTs) according to the preregistered exclusion criteria (more than 3 SDs slower than the sample mean in both the interrupting and take-over task). The final sample available for data analysis therefore consisted of *N* = 45 participants. Mean age was 23.1 years (SD = 3.7, range 18–33) and 37 participants were female. Participants reported to possess their driving license for on average 5.3 years (SD = 3.6, range 0.7–17). They indicated to drive on average 60.1 km per week (SD = 81.8, range 0–300).

### Tasks and stimuli

Videos of the driving scene were generated with SILAB software version 6.5^55^. The car drove from an Ego perspective on a road with four lanes. To reduce complexity, no other cars or road users were shown. Each video showed the car driving one of these four lanes (left, middle-left, middle-right, right) with one of four speeds (60 km/h, 80 km/h, 100 km/h, 120 km/h), to ensure sufficient complexity of the adopted speed and lane change task (see below for more information). The current speed was shown below the center of the screen (see Fig. 1). Thus, there were 16 combinations of driving scenes (4 lanes x 4 speeds). To increase variability, we created 4 versions of each combination, in which the video started on a different point of the road and the visual scenario therefore was slightly different, resulting in a total of 64 videos used. The videos were assigned to the different experimental conditions in a counter-balanced way, and the resulting trials were presented in randomized order.

**Fig. 4:**
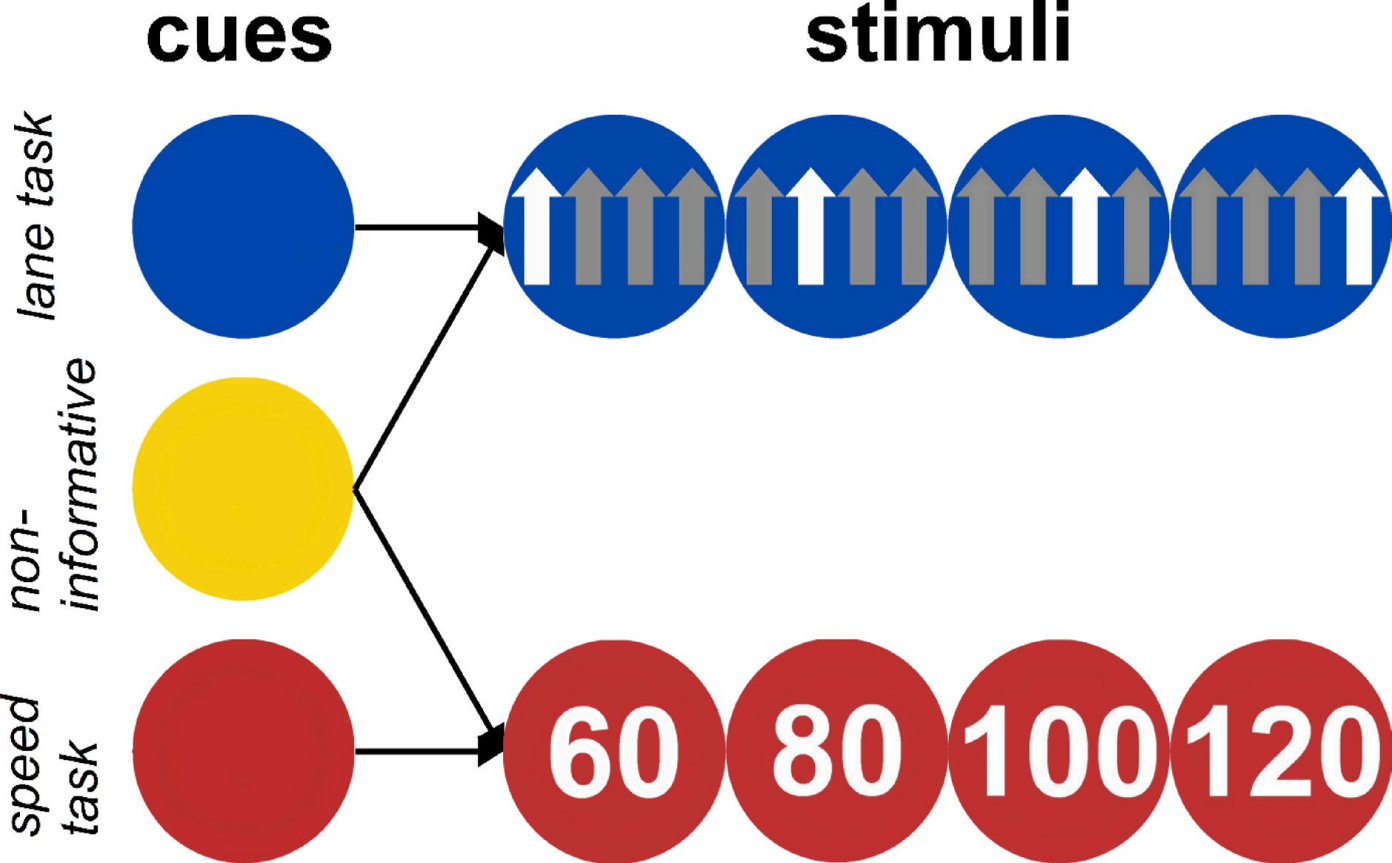
Overview of all cues and stimuli for the take-over task. Cues could either be informative (lane, speed task cue) or non-informative. For the former one, the cue predictively indicated the upcoming take-over task according to the color of the respective traffic sign of the stimuli. The color of the non-informative cue did not provide any signs for the upcoming task, and both speed and lane tasks followed non-informative cues with equal probability.

In each trial, participants watched such a video of the car autonomously driving the four-lane road (resolution 1280 × 1024 pixels, frame rate 60 Hz). After a randomly jittered duration (2, 3, 4, 5, 6, or 7 s), a cue was shown, which signaled either the upcoming lane or speed change task (conjointly referred to as take-over task) or was uninformative regarding the occurrence of these two tasks (for an overview of cues and stimuli, see Fig. 4).

Cues appeared at the center of the screen and were shown for 750 ms. Following the cue, either the take-over task (speed/lane change task) or the interrupting task (LDT) was presented, immediately after cue offset. For the interrupting LDT, participants had to classify letter strings into meaningful German words (e.g., “Bruder”, engl. “brother) or pronounceable, but meaningless pseudo words (e.g., “Ragter”). The letter string was shown in white ink at the center of the screen (all cues and stimuli were presented centrally). The LDT target remained on the screen until a response was given or until 2.5 s passed, which served as response deadline. If no response occurred until the deadline, “Zu langsam” (engl. “Too slow”) was shown for 300 ms in red. After the LDT, and an additional 300 ms in which no stimulus was shown (the video however was continuously played during the whole trial), the take-over task was presented, showing for each task one out of four stimuli (Fig. 4). The stimulus for the take-over task remained on the screen until response or a response deadline of 2.5 s, like for the LDT. If no interrupting task was presented, the take-over task immediately followed the cue. After each trial, i.e., after the response to the take-over task, a gray screen was shown for 1 s, followed by the next trial. The experiment was programmed and run in PsychoPy version v2023.2.1^56^.

Participants responded with one of two buttons of a response box. They pressed the left button with the index finger of their left hand, and the right button with the index finger of their right hand. For the interrupting LDT, the left or right buttons were pseudo-randomly assigned to the response categories “word” and “pseudoword” across participants. For the take-over task, response buttons were fixed. For the lane change task, a lane change to the left was assigned to the left button, and a lane change to the right to the right button. For the speed change task, an increase in speed was assigned to the right button, and a speed decrease to the left button. Likewise, in the take-over task, participants had to classify how the designated target speed or lane (the stimulus indicated the target lane or speed to which participants had change to) was associated with a change to the left/right or with an acceleration/deceleration with respect to their current lane/speed, respectively. For example, if the car drove on the middle-left lane with 80 km/h, and the speed task was shown with a stimulus indicating the participant to change speed to 60 km/h, participants had to press the left button to indicate a deceleration action.

In 80% of all trials (“main” trials), the car drove on the middle-left or middle-right lane with either 80 km/h or 100 km/h. For these trials, both speed and lane change task reflected a two-alternative forced-choice procedure, i.e., the stimuli could either afford a lane change to left or right, or an acceleration or deceleration of speed. For the other 20% of trials (“filler” trials), the car drove either on the outer left or right lane, and participants had to change one lane “inwards”, or the car drove with 60 km/h or 120 km/h, both scenarios uniquely affording an acceleration or deceleration action (indicated by an 80 km/h or 100 km/h stimulus, respectively). Accordingly, for filler trials only one meaningful response alternative was available. However, filler trials served to preclude that stimuli presented in main trials were only associated with one correct response alternative. For instance, without filler trials, the speed task stimulus “100 km/h” would have always been associated with an acceleration action (when the car drove with 80 km/h). However, in filler trials where the car drove with 120 km/h, the correct response to this stimulus was a deceleration action. Nevertheless, beyond preventing that participants respond to stimuli in main trials using learned stimulus-response associations, filler trials were of no further interest and excluded for analysis. For each participant, 192 main trials, and 48 filler trials were presented. For each trial type, speed and lane tasks occurred with equal probability, with these tasks being preceded by informative and non-informative equally often as well. The assignment of interrupting LDT stimuli, video version and cue onset jitter to the take-over tasks and responses were balanced.

### Procedure

The experiment was conducted in a dimly lit room. After arrival, participants performed the Ishihara color test and filled in questionnaires about their personal data and typical driving behavior. Afterwards, they performed a practice session. First, they practiced the LDT, the lane change and the speed change task in separate blocks. Afterwards, they performed the speed and lane change tasks together in one block without presence of the interrupting LDT. Finally, they performed a block of the experimental procedure with the interrupting LDT as presented in the main experiment. During the practice session, participants received trial-wise feedback about the correctness of their response. They could repeat practice blocks if they did not feel comfortable with the tasks yet.

Afterwards, the main experiment started. Instructions stressed both speed and accuracy of responses. The main experiment was divided into 8 blocks à 30 trials, between which participants could take a break. During these breaks, participants received feedback about their mean accuracy and response time in this block, separately for each task (LDT, lane change, speed change). However, no trial-wise feedback about the correctness of the response was provided in the main experiment. In total, the experimental procedures lasted for about 100 min.

### Statistical analysis

#### Data pre-processing

Response time (RT) and error rate (ER) data was only analyzed from main trials, i.e., filler trials, in which the procedure did not reflect a two-alternative forced-choice response procedure, were excluded from analysis. Furthermore, trials with no response until the response deadline as well as RT outliers were excluded (deviation of ± 2SD of an individuals mean RT^57,58^ for all analyses. For the analysis of RTs, only correct responses were considered, thus, in contrast to the analysis of ERs, here incorrect responses were additionally excluded. Lastly, regarding RTs and ERs in the take-over task, trials were also excluded if participants responded incorrectly or produced an RT outlier in the preceding interrupting LDT. After pre-processing, there remained on average 178 trials for the analysis of take-over task RTs (SD = 6, ~ 93% of main trials), and 180 trials for the analysis of ERs (SD = 5, ~ 94%). Regarding the analysis of the interrupting task, on average 89 trials remained for the analysis of RTs (SD = 4, ~ 93%) and 92 trials for the analysis of ERs (SD = 2, ~ 96%).

#### Conventional analysis of mean response times and error rates

For testing whether cue-informativity as well as the presence/absence of the interrupting task affected mean RTs/ERs, we calculated condition-wise averages of RTs and errors per participant. Regarding the analysis of performance in the interrupting LDT, trials in which the LDT was preceded by an informative vs. a non-informative cue were contrasted using a paired *t*-test. This analysis served to test whether specifically preparing for one of the two take-over tasks (i.e., informative cue condition) impeded interrupting task performance.

Concerning the analysis of performance in the take-over task, we calculated a repeated-measures ANOVA on mean RTs/ERs with the factors cue-informativity (informative vs. non-informative) and interrupting task (LDT absent vs. present), as well as the interaction of both factors. This was the main analysis of interest and served to test whether the informativity effect was reduced or reversed, when the cue was followed by an interrupting task. As typical for task switching analyses, analyses in the take-over task were aggregated across the speed and lane change tasks. All statistical analyses on mean RTs/ERs were performed using JASP^59^.

#### Effect course analysis

To track the time course of experimental effects, i.e., whether the modulation of the informativity effect in the take-over task by the absence/presence of the interrupting task changed with practice, we calculated effect course analyses^37^. We will only shortly outline this method here, for more detailed information the interested reader is referred to Berger, Kunde, & Kiefer^37^. In this method, RTs/ERs of each experimental condition are temporally ordered according to their occurrence in the experiment. Afterwards, they are smoothed by calculating moving averages, to reduce the influence of short-term fluctuations. Then, each (ordered) trial of each condition is contrasted against the respective trial of the other condition using a paired *t*-test. As this results in a large number of statistical comparisons, significance is assessed using a cluster-based permutation procedure^60^. Adjacent trials with a significant *t*-test are grouped together to clusters. Only the cluster *T*-value, that is the sum of all single *t*-values in this cluster, is subject to the statistical test of interest, i.e., the permutation test. During this permutation procedure, the labels of the experimental conditions are shuffled, and clusters are built like for the observed data as outlined above. Then, the *p*-value of an observed cluster is determined as the proportion of clusters in random permutations of the data exceeding this cluster’s *T*-value, i.e., indicating how unlikely it is to observe such a cluster by chance.

For the present analyses, the window size for calculation of moving averages was set to 11 trials, which corresponds to a fifth of the condition-wise available trials similar to our previous work^22,37^. Sample alpha was set to α = 0.1 and 5000 random permutations were run. We first calculated effect course analyses for the cue-informativity effect separately in trials in which the interrupting LDT was presented or was absent. Subsequently, to assess the course of the modulation of the informativity effect by the presence/absence of the interrupting LDT, these two effect courses were subtracted, and effect course analysis was performed on this data to assess the time course of the interaction effect.

#### Supplementary analyses

We performed additional supplementary analyses, reported in the *Supplementary Material*. First, we performed an effect course analysis also on ER data in the take-over task. Similar to the effect course analysis of RTs, this analysis showed a reduced informativity effect following the interrupting task around one third of the experiment. Second, effect course analyses were calculated on RT and ER data in the interrupting task (LDT), which showed the slowing in the LDT following informative cues to be reduced with practice. Third, we report effect course analyses on the effect of the interrupting task separately on take-over task performance following informative and non-informative cues. These analyses showed an effect of the presence/absence of the interrupting LDT on take-over task performance mainly for non-informative cues, reflected by significant slower take-over task RTs in this condition if no LDT was presented around one third of the experiment’s duration. Furthermore, we performed drift-diffusion model analyses^44,45,61^ of take-over task performance as well as interrupting LDT performance. Drift-diffusion model analyses in the take-over task again revealed a modulation of the cue-informativity effect by the interrupting task, with informative cues only being superior when not interrupted. Corresponding analyses on interrupting task performance showed a slowing in the LDT following informative cues on the non-decisional component, presumably aligning with interpretations that this component may also reflect switch-like processes^62^. We also present an extended discussion of the cognitive mechanisms probably causing the observed result pattern (*Supplementary Material F*), where we integrate the results of the drift-diffusion model analyses outlined above. Lastly, we report post hoc tests for the ANOVA analysis of take-over task RTs, to provide full information regarding which conditions differed from each other.

## Supplementary Information

Below is the link to the electronic supplementary material.

Supplementary Material 1

## Data Availability

The data and scripts to re-produce the analyses reported in the present work were uploaded to the Open Science Framework and are publicly available at: [https://osf.io/hg73v/](https:/osf.io/hg73v) .

## References

[1] SAE International. Taxonomy and definitions for terms related to driving automation systems for on-road motor vehicles. (2021). 10.4271/J3016_202104

[2] Shi, E. & Frey, A. T. Non-driving-related tasks during level 3 automated driving phases—Measuring what users will be likely to do. *Technol. Mind Behav.* **2**, 1 (2021).

[3] Vogelpohl, T., Kühn, M., Hummel, T., Gehlert, T. & Vollrath, M. Transitioning to manual driving requires additional time after automation deactivation. *Transp. Res. Part. F Traffic Psychol. Behav.* **55**, 464–482 (2018).

[4] Vogelpohl, T., Gehlmann, F. & Vollrath, M. Task interruption and control recovery strategies after Take-Over requests emphasize need for measures of situation awareness. *Hum. Factors*. **62**, 1190–1211 (2020). 31403839 10.1177/0018720819866976

[5] Zeeb, K., Buchner, A. & Schrauf, M. Is take-over time all that matters? The impact of visual-cognitive load on driver take-over quality after conditionally automated driving. *Accid. Anal. Prev.* **92**, 230–239 (2016). 27107472 10.1016/j.aap.2016.04.002

[6] Yoon, S. H., Kim, Y. W. & Ji, Y. G. The effects of takeover request modalities on highly automated car control transitions. *Accid. Anal. Prev.* **123**, 150–158 (2019). 30503824 10.1016/j.aap.2018.11.018

[7] Huang, G. & Pitts, B. J. Takeover requests for automated driving: The effects of signal direction, lead time, and modality on takeover performance. *Accid. Anal. Prev.* **165**, 106534 (2022). 34922107 10.1016/j.aap.2021.106534

[8] Miller, J. A., Nikan, S. & Zaki, M. H. Navigating the handover: Reviewing takeover requests in level 3 autonomous vehicles. *IEEE Open. J. Veh. Technol.* **5**, 1073–1087 (2024).

[9] Deng, H. et al. How to design driver takeover request in real-world scenarios: A systematic review. *Transp. Res. Part. F Traffic Psychol. Behav.* **104**, 411–432 (2024).

[10] Baumann, M. R. K., Petzoldt, T., Groenewoud, C., Hogema, J. & Krems, J. F. The effect of cognitive tasks on predicting events in traffic. In: *Proceedings of the European Conference on Human Centred Design for Intelligent Transport Systems* 3–11 (2008).

[11] De Winter, J. C. F., Happee, R., Martens, M. H. & Stanton, N. A. Effects of adaptive cruise control and highly automated driving on workload and situation awareness: A review of the empirical evidence. *Transp. Res. Part. F Traffic Psychol. Behav.* **27**, 196–217 (2014).

[12] Samuel, S., Borowsky, A., Zilberstein, S. & Fisher, D. L. Minimum time to situation awareness in scenarios involving transfer of control from an automated driving suite. *Transp. Res. Rec*. **2602**, 115–120 (2016).

[13] Endsley, M. R. Situation awareness misconceptions and misunderstandings. *J. Cogn. Eng. Decis. Mak.* **9**, 4–32 (2015).

[14] Endsley, M. R. Toward a theory of situation awareness in dynamic systems. *Hum. Factors*. **37**, 32–64 (1995).

[15] Baumann, M. R. K., Krems, J. F. A. Comprehension based cognitive model of situation awareness. *Lect Notes Comput. Sci. (including Subser. Lect Notes Artif. Intell. Lect Notes Bioinformatics)*. **5620 LNCS**, 192–201 (2009).

[16] Heenan, A., Herdman, C. M., Brown, M. S. & Robert, N. Effects of conversation on situation awareness and working memory in simulated driving. *Hum. Factors*. **56**, 1077–1092 (2014). 25277018 10.1177/0018720813519265

[17] Kass, S. J., Cole, K. S. & Stanny, C. J. Effects of distraction and experience on situation awareness and simulated driving. *Transp. Res. Part. F Traffic Psychol. Behav.* **10**, 321–329 (2007).

[18] Huang, G., Steele, C., Zhang, X. & Pitts, J. B. Multimodal cue combinations: A possible approach to designing in-vehicle takeover requests for semi-autonomous driving. *Proc. Hum. Factors Ergon. Soc.* **63**, 1739–1743 (2019).

[19] Petermeijer, S., Bazilinskyy, P., Bengler, K. & de Winter, J. Take-over again: Investigating multimodal and directional TORs to get the driver back into the loop. *Appl. Ergon.* **62**, 204–215 (2017). 28411731 10.1016/j.apergo.2017.02.023

[20] Wright, T. J. et al. Effects of alert cue specificity on situation awareness in transfer of control in level 3 automation. *Transp. Res. Rec*. **2663**, 27–33 (2017).

[21] Merlhiot, G. & Bueno, M. How drowsiness and distraction can interfere with take-over performance: A systematic and meta-analysis review. *Accid. Anal. Prev.* **170**, 106536 (2022). 34969510 10.1016/j.aap.2021.106536

[22] Berger, A., Koch, I. & Kiefer, M. Inhibition of cued but not executed task sets depends on cue-task compatibility and practice. *Psychol. Res.* **88**, 2036–2058 (2024). 39080024 10.1007/s00426-024-02013-z PMC11450066

[23] Berger, A. & Kiefer, M. Task set reconfiguration following masked and unmasked task cues. *Conscious. Cogn.* **130**, 103850 (2025). 40112356 10.1016/j.concog.2025.103850

[24] Hirsch, P., Koch, I. & Grundgeiger, T. Task interruptions. *Handb. Hum. Multitask.*. 10.1007/978-3-031-04760-2_4 (2022).

[25] Lenartowicz, A., Yeung, N. & Cohen, J. D. No-go trials can modulate switch cost by interfering with effects of task Preparation. *Psychol. Res.* **75**, 66–76 (2011). 20473686 10.1007/s00426-010-0286-3 PMC3016209

[26] Brass, M. & Von Cramon, D. Y. Decomposing components of task Preparation with functional magnetic resonance imaging. *J. Cogn. Neurosci.* **16**, 609–620 (2004). 15165351 10.1162/089892904323057335

[27] Swainson, R., Martin, D. & Prosser, L. Task-switch costs subsequent to cue-only trials. *Q. J. Exp. Psychol.* **70**, 1453–1470 (2017). 10.1080/17470218.2016.1188321 27174655

[28] Kiefer, M., Trumpp, N. M., Schaitz, C., Reuss, H. & Kunde, W. Attentional modulation of masked semantic priming by visible and masked task cues. *Cognition* **187**, 62–77 (2019). 30836302 10.1016/j.cognition.2019.02.013

[29] Kiesel, A. et al. Control and interference in task switching-A review. *Psychol. Bull.* **136**, 849–874 (2010). 20804238 10.1037/a0019842

[30] Monsell, S. Task switching. *Trends Cogn. Sci.* **7**, 134–140 (2003). 12639695 10.1016/s1364-6613(03)00028-7

[31] Arbuthnott, K. D. & Woodward, T. S. The influence of cue-task association and location on switch cost and alternating-switch cost. *Can. J. Exp. Psychol.* **56**, 18–29 (2002). 11901958 10.1037/h0087382

[32] Gade, M. & Steinhauser, M. The impact of cue format and cue transparency on task switching performance. *Psychol. Res.* **84**, 1346–1369 (2020). 30725390 10.1007/s00426-019-01150-0

[33] Grange, J. A. & Houghton, G. Cue-switch costs in task-switching: Cue priming or control processes? *Psychol. Res.* **74**, 481–490 (2010). 20037766 10.1007/s00426-009-0270-y

[34] Jost, K., De Baene, W., Koch, I. & Brass, M. A review of the role of cue processing in task switching. *Zeitschrift fur Psychologie / J. Psychol.* 221 5–14 (2013). 10.1027/2151-2604/a000125

[35] Berger, A., Kunde, W. & Kiefer, M. Task cue influences on lexical decision performance and masked semantic priming effects: The role of cue-task compatibility. *Atten. Percept. Psychophys*. **84**, 2684–2701 (2022). 36127490 10.3758/s13414-022-02568-2 PMC9630217

[36] Rydström, A., Mullaart, M. S., Novakazi, F., Johansson, M. & Eriksson, A. Drivers’ performance in Non-critical Take-Overs from an automated driving System—An On-Road study. *Hum. Factors*. **65**, 1841–1857 (2023). 35212565 10.1177/00187208211053460

[37] Berger, A., Kunde, W. & Kiefer, M. Dynamics of task Preparation processes revealed by effect course analysis on response times and error rates. *Sci. Rep.* **14**, 1–16 (2024). 38378818 10.1038/s41598-024-54823-1 PMC10879509

[38] Ansorge, U., Horstmann, G. & Worschech, F. Attentional capture by masked colour singletons. *Vis. Res.* **50**, 2015–2027 (2010). 20659496 10.1016/j.visres.2010.07.015

[39] Burnham, B. R. & Neely, J. H. A static color discontinuity can capture Spatial attention when the target is an abrupt-onset Singleton. *J. Exp. Psychol. Hum. Percept. Perform.* **34**, 831–841 (2008). 18665729 10.1037/0096-1523.34.4.831

[40] Horstmann, G. Evidence for attentional capture by a surprising color Singleton in visual search. *Psychol. Sci.* **13**, 499–505 (2002). 12430832 10.1111/1467-9280.00488

[41] Koch, I., Gade, M., Schuch, S. & Philipp, A. M. The role of Inhibition in task switching: A review. *Psychon Bull. Rev.* **17**, 1–14 (2010). 20081154 10.3758/PBR.17.1.1

[42] Pashler, H. Dual-task interference in simple tasks: Data and theory. *Psychol. Bull.* **116**, 220–244 (1994). 7972591 10.1037/0033-2909.116.2.220

[43] Koch, I., Poljac, E., Müller, H. & Kiesel, A. Cognitive structure, flexibility, and plasticity in human multitasking-an integrative review of dual-task and task-switching research. *Psychol. Bull.* **144**, 557–583 (2018). 29517261 10.1037/bul0000144

[44] Ratcliff, R. & McKoon, G. The diffusion decision model: Theory and data for two-choice decision tasks. *Neural Comput.* **20**, 873–922 (2008). 18085991 10.1162/neco.2008.12-06-420 PMC2474742

[45] Voss, A., Nagler, M. & Lerche, V. Diffusion models in experimental psychology: A practical introduction. *Exp. Psychol.* **60**, 385–402 (2013). 23895923 10.1027/1618-3169/a000218

[46] Wandtner, B., Schömig, N. & Schmidt, G. Effects of Non-Driving related task modalities on takeover performance in highly automated driving. *Hum. Factors*. **60**, 870–881 (2018). 29617161 10.1177/0018720818768199

[47] Dreisbach, G. Mechanisms of cognitive control: The functional role of task rules. *Curr. Dir. Psychol. Sci.* **21**, 227–231 (2012).

[48] Dreisbach, G. & Haider, H. That’s what task sets are for: Shielding against irrelevant information. *Psychol. Res.* **72**, 355–361 (2008). 18057961 10.1007/s00426-007-0131-5

[49] Scheil, J. & Kleinsorge, T. N. – 2 repetition costs depend on Preparation in trials n – 1 and n – 2. *J. Exp. Psychol. Learn. Mem. Cogn.* **40**, 865–872 (2014). 24364724 10.1037/a0035281

[50] Scheil, J. & Kleinsorge, T. Effects of a dynamically changing response set overlap on n – 2 repetition costs. *Psychol. Res.* **1**, 1–8 (2023). 10.1007/s00426-023-01816-w PMC10457239 36949242

[51] Scheil, J. & Kleinsorge, T. Inhibition during task switching is affected by the number of competing tasks. *Mem. Cogn.* **52**, 211–224 (2024). 10.3758/s13421-023-01456-w PMC10805815 37698800

[52] Miller, J. How many participants? How many trials? Maximizing the power of reaction time studies. *Behav. Res. Methods*. 10.3758/S13428-023-02155-9 (2023). 37537492 10.3758/s13428-023-02155-9 PMC10991062

[53] Janssen, C. P., Iqbal, S. T., Kun, A. L. & Donker, S. F. Interrupted by my car? Implications of interruption and interleaving research for automated vehicles. *Int. J. Hum. Comput. Stud.* **130**, 221–233 (2019).

[54] Chai, C. et al. The effects of various auditory takeover requests: A simulated driving study considering the modality of non-driving-related tasks. *Appl. Ergon.* **118**, 104252 (2024). 38417230 10.1016/j.apergo.2024.104252

[55] Würzburger Institute for Traffic Science GmbH. Driving simulation and SILAB. at. (2014).

[56] Peirce, J. et al. PsychoPy2: Experiments in behavior made easy. *Behav. Res. Methods*. **2019 511 51**, 195–203 (2019). 10.3758/s13428-018-01193-y PMC6420413 30734206

[57] Berger, A. & Kiefer, M. Comparison of different response time outlier exclusion methods: A simulation study. *Front. Psychol.* **12**, 675558 (2021). 34194371 10.3389/fpsyg.2021.675558 PMC8238084

[58] Berger, A. & Kiefer, M. Electrophysiological correlates of response time outliers: Outlier related potentials. *Psychophysiology* **60**, e14305 (2023). 37042066 10.1111/psyp.14305

[59] JASP Team. JASP (Version 0.14.1)[Computer software]. at (2020). https://jasp-stats.org/

[60] Maris, E. & Oostenveld, R. Nonparametric statistical testing of EEG- and MEG-data. *J. Neurosci. Methods*. **164**, 177–190 (2007). 17517438 10.1016/j.jneumeth.2007.03.024

[61] Ratcliff, R. A theory of memory retrieval. *Psychol. Rev.* **85**, 59–108 (1978).

[62] Schmitz, F. & Voss, A. Decomposing task-switching costs with the diffusion model. *J. Exp. Psychol. Hum. Percept. Perform.* **38**, 222–250 (2012). 22060144 10.1037/a0026003

