# Supplementary material for: The role of take-over cue informativity in interrupted take-over requests in a semi-automated driving scenario
Source: Sci Rep. 2026 Jan 20;16:2628. doi: 10.1038/s41598-026-36614-y (PMC12824352; doi:10.1038/s41598-026-36614-y)
Supplement: Supplementary file 1 — Supplementary Material 1 [file 41598_2026_36614_MOESM1_ESM.pdf]

Supplementary Material

The role of take-over cue informativity in interrupted take-over requests in a semi-automated driving scenario

Alexander Berger[1*], Nicole Damm[2], Martin Baumann[2], and Markus Kiefer[1]

[1]Ulm University, Department of Psychiatry, Ulm, Germany

[2]Ulm University, Department of Psychology, Ulm, Germany

A: Effect course analysis of ERs in the take-over task 2

B: Effect course analyses of RTs and ERs in the interrupting task........ 4

C: Effect course analyses of the influence of the interrupting task on take-over task performance separately per cue-informativity conditions........ 5

D: Drift-diffusion model analyses of take-over task performance........ 7

E: Drift-diffusion model analyses of interrupting task performance 9

F: Discussion of cognitive mechanisms causing the observed modulation of the cue-informativity effect by the presence/absence of the interrupting task........ 10

G: Post hoc tests for take-over task response times 12

Supplementary references 13

A: Effect course analysis of ERs in the take-over task

Similar to the corresponding analysis reported in the main manuscript, we performed effect course analyses (Berger, Kunde, et al., 2024) on ERs in the take-over task. We analyzed the informativity effect separately in trials where the interrupting task was absent and present, as well as the difference between these two effect courses, i.e., the interaction of cue-informativity and interrupting task.

Neither for the informativity effect in trials where the interrupting LDT was absent, nor in trials where this LDT was presented, effect course analysis revealed a significant cluster. However, we observed a significant cluster for the interaction effect, $T = -19.77$, $p = .025$, trials 10 – 17. Similar to the analysis of RTs, this indicated a larger informativity effect in trials where the interrupting task was absent (*Figure S1c*), which can be interpreted as a consequence of the LDT interfering with preparation for the take-over task. As for the analysis of RTs, this modulation vanished with further practice.

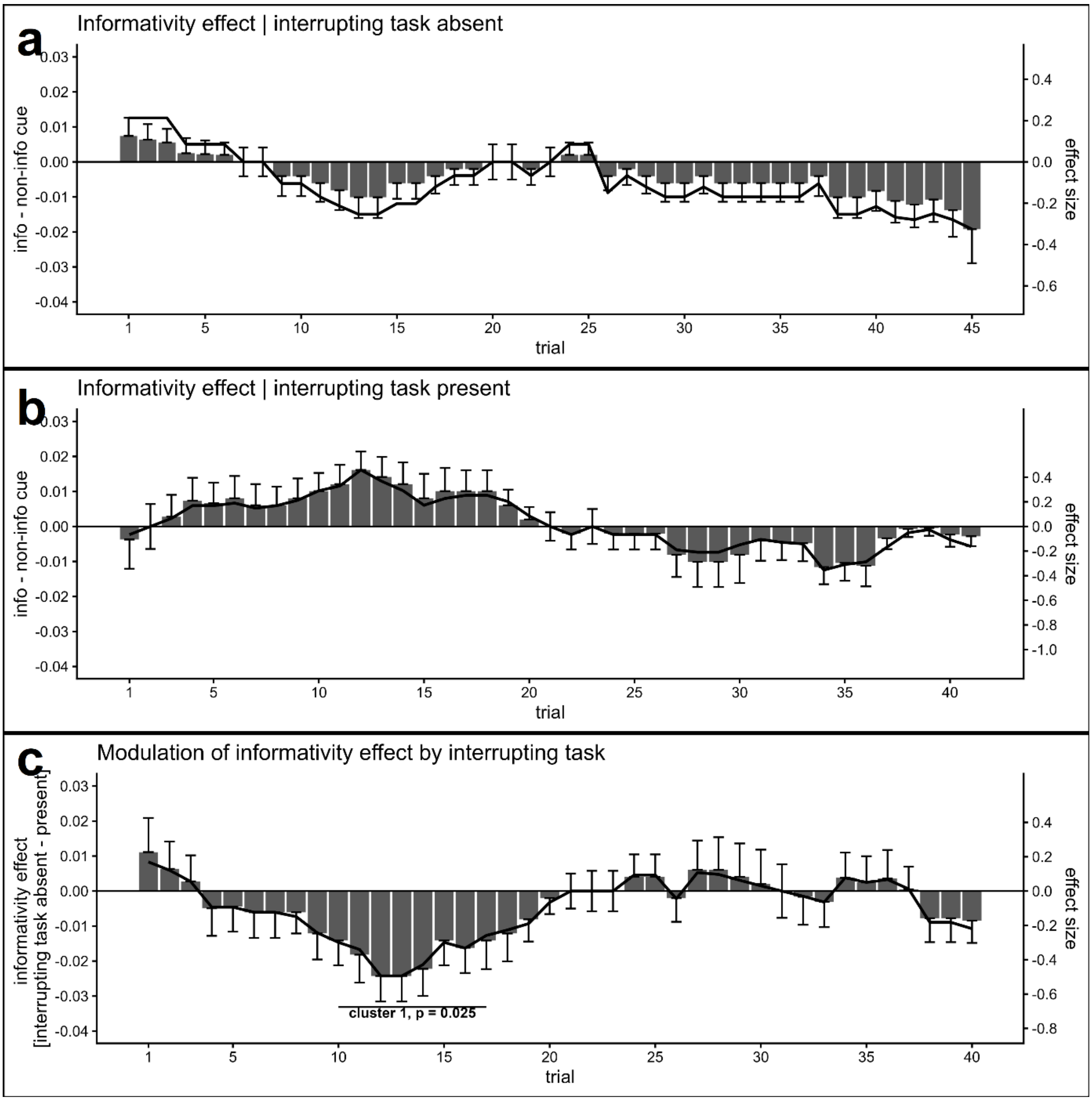

Figure S1: Effect course analyses on take-over task ERs. The bars show the ER difference (plus associated SEs as whiskers) between informative and non-informative cues separately for trials where no LDT was presented, and which were preceded by the interrupting LDT (panel a and b, respectively). The black line shows the effect size (Cohen's d). Panel c depicts the difference between these two effect courses, i.e., the modulation of the informativity effect by the absence/presence of the interrupting task. Similar for the effect course analysis of take-over task RTs, a significant cluster for the interaction was only observed in the first half of the experiment, after some initial practice.

B: Effect course analyses of RTs and ERs in the interrupting task

Effect course analyses of performance in the interrupting LDT as a function of cue informativity revealed one significant cluster for the analysis of RTs, $T = 111.01$, $p < .001$, trials 1 – 29. RTs in the interrupting task were slower, if preceded by an informative cue. However, this effect was reduced with practice and could be no longer observed at the end of the experiment (see *Figure S2*). Accordingly, effects of preparation elicited by TOR cues on the interrupting task vanished with practice. A corresponding effect course analysis on ERs in the interrupting LDT showed no significant cluster.

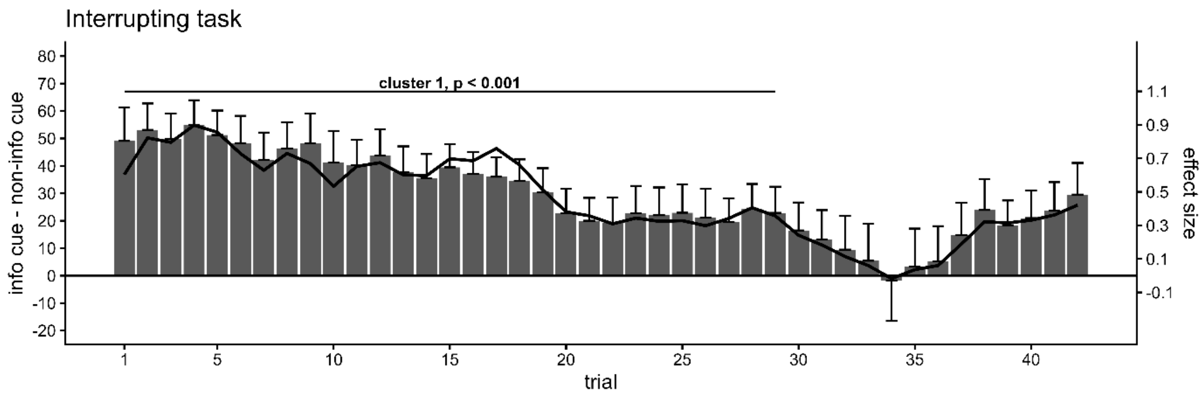

Figure S2 Effect course analysis of RTs in the interrupting LDT.

C: Effect course analyses of the influence of the interrupting task on take-over task performance separately per cue-informativity conditions

To get additional insights into which specific conditions caused the observed modulation of the cue-informativity effect by the interrupting task, we conducted another effect course analysis on take-over task RTs. While the informativity effect was reduced following the presentation of an interrupting task (see the main text), averaged RTs (*Figure 2* in the main text) indicated that this might be a consequence of the RTs following non-informative cues being elevated, if the interrupting task lacked. To this end, we analyzed the effect course of the absence/presence of the interrupting LDT, separately for informative and non-informative cues.

In trials in which an informative cue was presented, effect courses analysis revealed no significant cluster, and there were no pronounced differences in take-over task performance regardless of whether the informative cue was followed by the interrupting LDT or not (*Figure S3a*). However, for trials including a non-informative cue, an effect of the interrupting task was observed. RTs in the take-over task were slower, if the interrupting LDT lacked compared to when it was presented, $T = 44.02$, $p = .004$, trials 8 – 23. Thus, participants responded *slower*, if no interruption occurred, but only after some practice and not at the experiment's end, where this effect had vanished (*Figure S3b*). This slowing following the absence of a LDT corresponded temporally with the observed cluster for the interaction effect, $T = -51.65$, $p < .001$, trials 7 – 23 (*Figure S3c*). Note that this cluster naturally was the same as observed in the corresponding analysis reported in the main text (*Figure 3c*), as we only changed the order of comparison in the present analysis.

Hence, this analysis supports that effects of preparation were only present in the first half of the experiment, with these effects being mainly reflected in the RT in trials with a non-informative cue. After further practice, a strategy change occurred or interferences were reduced, causing a further absence of this modulation. For a discussion why effects of the interrupting task mapped on the non-informative cue condition, see *Supplementary Material F* below.

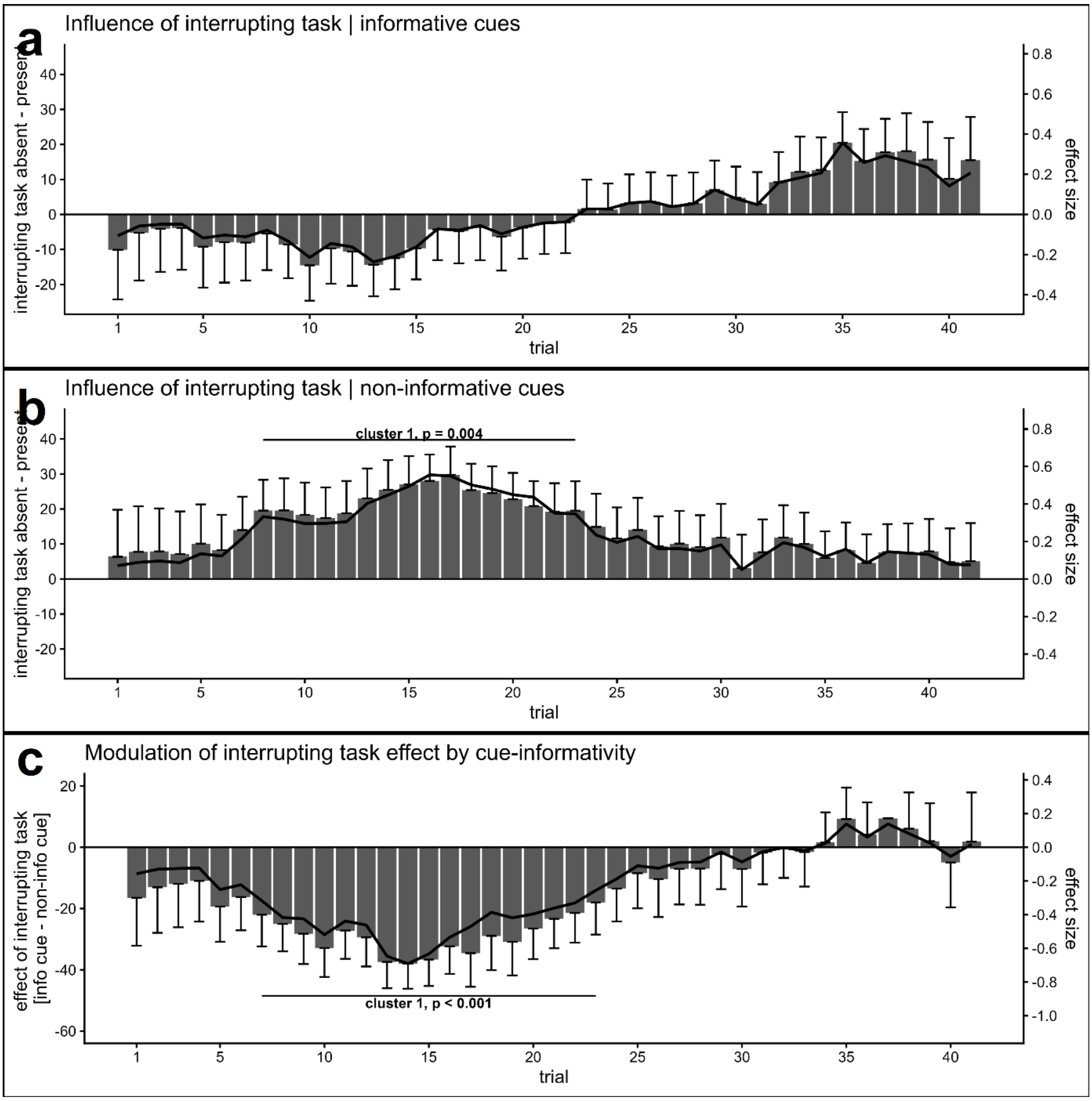

Figure S3 Effect course analyses of take-over task RT for the effect of the interrupting task depending on the type of cue. Shown are the effect courses of the absence/presence of the interrupting LDT separately for trials with an informative cue (panel a), a non-informative cue (panel b) as well as the interaction of these two conditions (panel c).

D: Drift-diffusion model analyses of take-over task performance

To obtain additional insights regarding the cognitive processes involved in task performance in the present study, we conducted drift-diffusion model analyses (Ratcliff, 1978; Ratcliff & McKoon, 2008). Drift-diffusion model analyses conjointly integrate single-trial RT and ER data and decompose this data into three main parameters, which are linked to specific cognitive processes (Voss et al., 2013). The drift rate *v* reflects the speed of information processing, while the decision threshold *a* represents the amount of information needed for a response threshold to be reached, i.e., response caution. Other processes outside the decision process like stimulus encoding or response production are usually mapped to the non-decision time *t0*.

In the present study, we defined correct and incorrect responses as response alternatives, and fixed the starting parameter to *a*/2, i.e., reflecting no bias between correct and incorrect responses. Drift-diffusion models were estimated in a hierarchical Bayesian framework using the HDDM toolbox in Python (Wiecki et al., 2013). We used 10,000 samples with a burn-in of 3000. Model convergence was assessed visually and evaluated as satisfactory. For the analysis of the take-over task, the drift rate, the decision threshold and the non-decision time were allowed to vary for cue-informativeness and interrupting task (LDT absent vs. present). Mean drift-diffusion model parameters per subject were extracted and handed to a Bayesian repeated-measures ANOVA as implemented in JASP (JASP Team, 2020). Evidence for an effect was assessed using Bayes Factors following the guidelines by Wagenmakers et al. (2017).

Mean drift-diffusion model parameters averaged across participants are shown in *Table S1*. For drift rates, there was no evidence for an effect of cue-informativity (BF = 0.19), but extreme evidence for an effect of interrupting task (BF = 1413.95) and the interaction of both effects (BF = 3056.45). Drift rates in the take-over task were on average larger, if not preceded by an interrupting LDT. Moreover, drift rates were only larger for informative compared to non-informative cues, if the interrupting task was absent. Otherwise, following the interrupting task, drift rates were larger for non-informative compared to informative cues.

Regarding the decision threshold, there was neither compelling evidence for an effect of cue-informativity (BF = 0.31) nor of interrupting task (BF = 0.78). However, there was extreme evidence for an interaction of both factors (BF = 2.8*10^11). When the interrupting task was absent, decision thresholds were elevated for informative compared to non-informative cues. In contrast, following an interrupting task, decision thresholds were smaller for informative than non-informative cues.

Lastly, for non-decision times, there was extreme evidence for an effect of cue-informativity (BF = 1.9*10^9) and very strong evidence for an effect of interrupting task (BF = 35.2). Furthermore, the interaction of both factors reached extreme evidence as well (BF = 2.9*10^15). On average, non-decision times in the take-over task were elevated for non-informative cues, as well as when the interrupting LDT was absent. However, these effects were mainly driven by largely elevated non-decision times following non-informative cues, when the interrupting task was absent.

In summary, drift-diffusion model analyses in the take-over task showed interference effects between the interrupting and take-over task, indicated by lower drift rates when the take-over task was preceded by the interrupting task. Moreover, drift rates and decision thresholds indicated more efficient processing (drift rates) and cautious responding (decision thresholds) following informative cues, but only when no interrupting task was presented. Otherwise

(presence of an interrupting task), processing/responding was more efficient/cautious for non-informative cues. These analyses suggest a modulation of the cue-informativity effect by the interrupting task, with informative cues only being superior when not interrupted by an interrupting task. Furthermore, the analysis of non-decision times showed an especially elevated *t0* following non-informative cues, when no interrupting task was presented. This could align with the interpretation that participants (somehow) used the non-informative cue to prepare for the interrupting task, leading to delayed responding if the take-over task immediately followed a non-informative cue (similar to switch costs, cf.; Schmitz & Voss, 2012).

Table S1 Mean drift-diffusion model parameters (+ SD in parentheses) in the take-over task.

		DDM parameter		
**Interrupting task**	**cue-informativity**	*drift rate*	*decision threshold*	*non-decision time*
absent	*informative*	4.39 (0.65)	2.44 (0.43)	0.30 (0.04)
	non-informative	4.14 (0.68)	2.21 (0.44)	0.37 (0.03)
present	*informative*	3.81 (0.56)	2.06 (0.41)	0.32 (0.03)
	non-informative	4.03 (0.69)	2.40 (0.43)	0.32 (0.03)

E: Drift-diffusion model analyses of interrupting task performance

Drift-diffusion model analyses in the interrupting LDT were performed similar to the above-outlined analyses of take-over task performance, with drift rates, decision thresholds and non-decision times varying for cue-informativity. However, as only one factor was tested, we performed Bayesian paired samples t-tests to assess evidence for the presence of an effect of cue-informativity.

These analyses revealed neither for drift rates (BF = 0.17), nor for decision thresholds (BF = 0.17) evidence for an effect of cue-informativity. In contrast, for non-decision times there was extreme evidence for such an effect (BF = 3445.54), showing longer non-decision times in the interrupting task if preceded by an informative cue (see *Table S2*). Hence, presenting an informative cue in advance of the interrupting LDT slowed responding, supporting the claim that participants used the non-informative cue to prepare for the interrupting LDT and/or that preparing for the take-over task interfered with LDT performance.

Table S2 Mean drift-diffusion model parameters (+ SD in parentheses) in the interrupting task.

	**DDM parameter**		
**cue-informativity**	*drift rate*	*decision threshold*	*non-decision time*
informative	3.05 (0.85)	1.81 (0.38)	0.49 (0.06)
non-informative	3.08 (0.84)	1.83 (0.40)	0.46 (0.06)

F: Discussion of cognitive mechanisms causing the observed modulation of the cue-informativity effect by the presence/absence of the interrupting task

Here we will present a discussion regarding the specific cognitive mechanisms which could have caused the observed results (i.e., reduction of the cue-informativity effect after an interrupting task). To summarize the main findings (see also the main text), task-specific informative cues aided performance in the take-over task in terms of faster RTs. This task-specific preparation elicited by informative cues was also reflected by worse performance in the interrupting LDT following an informative cue, suggesting that preparation for the take-over task interfered with the LDT and/or had to be actively suppressed when interrupted. From a mechanistic perspective, participants probably used the informative cue to allocate attention to information relevant for performing the cued task. For example, when a cue for the speed task was presented, participants could have focused on the current speed, which needed to be compared to the designated target speed indicated by the stimulus, to identify whether acceleration or deceleration was required. This attentional focus then interfered with processing the interrupting LDT, if the LDT followed an informative cue.

Crucially, the cue-informativity effect was reduced following the interrupting LDT, probably reflecting the inhibition of the prepared task set when faced with an interrupting task (Berger et al., 2022; Berger, Koch, et al., 2024; Kiefer et al., 2019). When inspecting this data pattern in more detail, this reduction of the informativity effect following the interrupting LDT was primarily driven by a modulation of RTs in the non-informative cue condition, while RTs after informative cues remained stable across interrupting task conditions (compare Figure 2 in the main text). As the general impact of the presence or absence of the interrupting LDT on the level of take-over task RT cannot be determined given the lack of a neutral condition, two alternative accounts are conceivable. First, participants might have prepared for a dual-task sequence, planning to execute the LDT before the take-over task. This would generally accelerate RTs when the expected sequence occurred (i.e., when the LDT was presented). After non-informative cues, this acceleration is observed, i.e., RTs are faster after the interrupting LDT than without LDT. For informative cues, such a strategy should have also resulted in faster responding when the interrupting LDT was presented. However, due to interference between the prepared task set of the take-over task and the LDT, the prepared task set was inhibited, masking such an expected net improvement in RT (task set inhibition effects should only affect the informative cue condition, where task-specific preparation was possible, cf. Koch et al., 2010). In line with this reasoning, drift-diffusion model analyses reported in *Supplementary Material D* showed an advantage of informative cues regarding processing speed and decision threshold only when no LDT was presented, but a disadvantage if informative cues were interrupted by the LDT.

On the other hand, participants could have used non-informative cues to prepare for the interrupting LDT as no specific take-over task was signaled (albeit the LDT following such cues only in 50% of all trials), whereas informative cues triggered only preparation for the cued take-over task. In this case, the absence of the LDT after a non-informative cue impaired take-over task performance, as participants had to switch from preparation for the LDT to the take-over task (compare *Supplementary Material D*, showing increased non-decision times under these conditions, likely reflecting switching costs). Stable performance following informative cues could suggest that the prepared task set might have been kept active during the LDT, facilitating take-over task performance independent of the interrupting LDT (Berger, Kunde, et al., 2024). Regardless of the specific mechanism, the reduced informativity effect following an

interrupting task underscores the impact of interference on task preparation, emphasizing the need to consider distractions in the context of TORs. Moreover, as suggested by practice-dependent effects observed in this study, the impact of such interferences may not be static.

In the light of the above-outlined cognitive mechanisms, these strategies probably also changed with practice. After some initial practice, participants might have recognized that following an informative cue, always the cued take-over task was shown (being interrupted by the LDT or not). In contrast, a non-informative cue was neutral regarding the identity of the later presented take-over task, probably encouraging participants to prepare more for the LDT following such cues. As a result, they were slower in the take-over task if it immediately followed a non-informative cue (see *Supplementary Material C*, which showed this slowing to overlap with the observed cluster for the modulation of the informativity effect). After further practice, they withdrew this strategy (as it is inefficient due to the LDT equally often being presented or not following all cue types), mitigating any effects of the presence/absence of the interrupting LDT (for a recent discussion on the dependence of adaptations of top-down control on practice, see Braem et al., 2024). Regarding the alternative account, i.e., that participants prepared for a dual-task sequence; with more practice within that dual-task sequence, interference between these two tasks probably was reduced. This might reflect a general shielding of task sets from interference (Dreisbach, 2012; Dreisbach & Haider, 2008) or an integration of two task sets into one over-arching set (Koch et al., 2018) with practice (see also the main text). Reduced interference between task sets consequently also reduces the need for task set inhibition (as there is less conflict between task sets), mitigating the effects of the interrupting task (cf., Koch et al., 2010).

G: Post hoc tests for take-over task response times

Table S3 Post hoc comparisons for the ANOVA on take-over task RTs.

		Mean Difference	*SE*	*t*	p_{holm}	*Cohen's d*
info cue, interrupting task absent	non-info cue, interrupting task absent	-50.4	6.4	-7.91	< .001	-0.65
	info cue, interrupting task present	-1.0	6.3	-0.17	0.869	-0.01
	non-info cue, interrupting task present	-37.6	7.8	-4.84	< .001	-0.49
non-info cue, interrupting task absent	info cue, interrupting task present	49.3	7.5	6.55	< .001	0.64
	non-info cue, interrupting task present	12.7	6.0	2.11	0.081	0.17
info cue, interrupting task present	non-info cue, interrupting task present	-36.6	6.0	-6.09	< .001	-0.48

Note. P-value adjusted for comparing a family of 6 estimates using the Holm method. All dfs = 44. All mean differences in ms.

Supplementary references

Berger, A., Koch, I., & Kiefer, M. (2024). Inhibition of cued but not executed task sets depends on cue-task compatibility and practice. *Psychological Research*, *88*(7), 2036–2058. https://doi.org/10.1007/S00426-024-02013-Z

Berger, A., Kunde, W., & Kiefer, M. (2022). Task cue influences on lexical decision performance and masked semantic priming effects: The role of cue-task compatibility. *Attention, Perception, and Psychophysics*, *84*(8), 2684–2701. https://doi.org/10.3758/S13414-022-02568-2

Berger, A., Kunde, W., & Kiefer, M. (2024). Dynamics of task preparation processes revealed by effect course analysis on response times and error rates. *Scientific Reports*, *14*(1), 1–16. https://doi.org/10.1038/s41598-024-54823-1

Braem, S., Chai, M., Held, L. K., & Xu, S. (2024). One cannot simply 'be flexible': regulating control parameters requires learning. *Current Opinion in Behavioral Sciences*, *55*, 101347. https://doi.org/10.1016/J.COBEHA.2023.101347

Dreisbach, G. (2012). Mechanisms of cognitive control: The functional role of task rules. *Current Directions in Psychological Science*, *21*(4), 227–231. https://doi.org/10.1177/0963721412449830

Dreisbach, G., & Haider, H. (2008). That's what task sets are for: Shielding against irrelevant information. *Psychological Research*, *72*(4), 355–361. https://doi.org/10.1007/S00426-007-0131-5

JASP Team. (2020). *JASP (Version 0.14.1)[Computer software]*. https://jasp-stats.org/

Kiefer, M., Trumpp, N. M., Schaitz, C., Reuss, H., & Kunde, W. (2019). Attentional modulation of masked semantic priming by visible and masked task cues. *Cognition*, *187*, 62–77. https://doi.org/10.1016/j.cognition.2019.02.013

Koch, I., Gade, M., Schuch, S., & Philipp, A. M. (2010). The role of inhibition in task switching: A review. *Psychonomic Bulletin and Review*, *17*(1), 1–14. https://doi.org/10.3758/PBR.17.1.1

Koch, I., Poljac, E., Müller, H., & Kiesel, A. (2018). Cognitive structure, flexibility, and plasticity in human multitasking-an integrative review of dual-task and task-switching research. *Psychological Bulletin*, *144*(6), 557–583. https://doi.org/10.1037/bul0000144

Ratcliff, R. (1978). A theory of memory retrieval. *Psychological Review*, *85*(2), 59–108. https://doi.org/10.1037/0033-295X.85.2.59

Ratcliff, R., & McKoon, G. (2008). The diffusion decision model: Theory and data for two-choice decision tasks. *Neural Computation*, *20*(4), 873–922. https://doi.org/10.1162/NECO.2008.12-06-420

Schmitz, F., & Voss, A. (2012). Decomposing task-switching costs with the diffusion model. *Journal of Experimental Psychology: Human Perception and Performance*, *38*(1), 222–250. https://doi.org/10.1037/A0026003

Voss, A., Nagler, M., & Lerche, V. (2013). Diffusion models in experimental psychology: a practical introduction. *Experimental Psychology*, *60*(6), 385–402. https://doi.org/10.1027/1618-3169/A000218

Wagenmakers, E.-J., Love, J., Marsman, M., Jamil, T., Ly, A., Verhagen, J., Selker, R., Gronau, Q. F., Dropmann, D., Boutin, B., Meerhoff, F., Knight, P., Raj, A., van Kesteren, E.-J., van Doorn, J., Šmíra, M., Epskamp, S., Etz, A., Matzke, D., … Morey, R. D. (2017). Bayesian inference for psychology. Part II: Example applications with JASP. *Psychonomic Bulletin & Review 2017 25:1*, *25*(1), 58–76. https://doi.org/10.3758/S13423-017-1323-7

Wiecki, T. V, Sofer, I., & Frank, M. J. (2013). HDDM: Hierarchical Bayesian estimation of the Drift-Diffusion Model in Python. *Frontiers in Neuroinformatics*, *0*(JULY 2013), 14. https://doi.org/10.3389/FNINF.2013.00014
